# Uterine Displacements

**Published:** 1869-01-01

**Authors:** H. Webster Jones

**Affiliations:** Accoucheur to Cook Co. Hospital


### UTERINE DISPLACEMENTS.

BY H. WEBSTER JONES, A.M., M.D., ACCOUCHEUR TO COOK CO. HOSPITAL.

FOUR years ago, when commenting in this JOURNAL upon a certain method of treatment previously proposed for these disorders by another, the writer took occasion to enlarge upon means apparently more desirable, contenting himself, however, in simple statement, without illustration or proof.

Since that time, the results of experience, and my observation of the theory and practice of others, as well as the direct queries of many, have more abundantly conduced to the opinion, first, that dislocations of the uterus are underrated, in respect to their bearing upon the comfort and general health of patients; second, that the natural supports of the organ are not fairly understood in their comparative and mutual relations; and, third, that the difficulties of adjustment, in a great variety of cases, have disheartened some, and altogether deterred others from the use of proper mechanical aids.

It is my present purpose, therefore, to narrate a few cases, illustrating the importance of uterine deviations, whether greater or less in degree, and the need of more careful examinations by medical men, lest these ailments escape the attention they deserve.

Dr. Tilt, in his work on "Uterine Therapeutics," relates the case of a young married lady, who consulted him on account of "continued pain in the sacrum and loins, with bearing down pains increased upon the slightest exertion, so that walking was intolerable to her."

Her sufferings had commenced soon after a labor, two years previous. Many persons had been consulted, but they found no organic lesion or deviation. Cold douches, sedatives, and injections gave little relief. When examined, in the standing posture, the womb did not appear prolapsed or deviated, but on raising it upon the tips of two fingers, the patient exclaimed, "You have taken away all my suffering!" "An air-pessary was applied, which gave permanent relief, and was worn for six weeks, after which time the patient was able to discontinue its use."

Dr. Debout, editor of the "Bulletin Therapeutique," mentions an unmarried lady, who had been reduced to the last stages of inanition by continued abdominal pains.

Several of the first obstetric authorities of Paris, when consulted, had detected "no uterine lesion or displacement," and their suggestions were practically futile. Dr. Debout examined the patient standing, lifted the uterus, and the patient exclaimed at the relief afforded. In this case, also, the air-pessary "removed the pains, permitted food to be taken, and sleep to be enjoyed." "Supporting and steadying the womb, its nerves soon lost a habit of suffering, though the neuralgia had persisted for years."

In August last I was asked to see a lady, who understood that I "had something new for womb-complaint."

She had taken the advice of eminent men, one of whom diagnosed cervical hypertrophy, and proposed amputation. Alarmed, she placed herself under the care of a female practitioner, who cured her of slight ulcerations, and pronounced her "as well as she ever could be." But she stated that her back had ached "most of the time for five years," and at the present time "to walk for half an hour was certain exhaustion, and to sit or stand as long was misery."

An examination of this case disclosed a uterus prolapsed in the axis of the pelvic outlet; a mucous membrane, healthful every where, and a vagina so relaxed as that, when the finger was passed around the cervix, it was very difficult to say where the neck ended, or the vagina began.

The womb was replaced and supported upon a closed-bar lever pessary, and the patient started the same night upon a journey of five hundred miles. A month afterward she reported that she had never traveled with so great comfort, and that "it was ecstacy to be without a back-ache." This relief has continued to the present time.

In October it was my province to treat a case of supposed lumbago and sciatica, occurring in a young unmarried lady. It was her first experience of the kind, and it had commenced soon after a misstep in descending from a table, upon which she had mounted to re-adjust a window shade. After ten days of vain attempts with medicines and external applications, her prone posture in walking, pain in the right loin, and sensation, as of a moving body, within the pelvis, upon any effort at locomotion, led me to examine the uterus. It was prolapsed, and rested upon the rectum. A pessary was adjusted, medicine discontinued, and in less than a day the patient was, in her own words, "as well as ever."

In the cases already related, the symptoms were of so localized a nature as to lead, at an early period, to the suspicion of uterine disorder. But one occasionally meets with an example of a different sort, of which the following may serve as an illustration:

Early in 1843, a lady from an adjoining town applied to me for relief from an oft-recurring colic, which, seated in the epigastric region, was occasionally so intense as to rob her of reason.

It had been supposed, by her medical advisers, that this pain was due to biliary calculi, but appropriate treatment had proven unavailing, and the patient had for five months been unable to walk, even a few rods at a time, without premonitions of colic.

It occurred *always* after an attempt at sweeping her rooms. She dated the first attack soon after a miscarriage at four months, which had taken place two years previous to our interview.

She presented the aspect of perfect health, menstruated normally, and had noticed no increased tendency to suffer at such periods. She had never had leucorrhœa, and rarely, if ever, back-ache, and I could with difficulty induce her to submit to a vaginal examination, so convinced was she that she had only "liver complaint."

By a digital examination (ocular inspection was not allowed), made in a standing posture, the uterus was found to be completely retroverted, and somewhat enlarged. The pessary of Dr. Hodge was advised and introduced. Two weeks afterward, a larger one was substituted, which remained in situ for about a year, during which time she had not experienced a single attack of colic. I have learned, within a few days, of her remaining in perfect health.

Cases of similar import are not of infrequent occurrence in the writer's experience, but enough has now been said to establish, first, that *much suffering may depend upon the existence of a greater or less degree of uterine displacement;* second, that *such suffering may be hopefully treated by mechanical means, addressed to the erring organs.*

It is, moreover, intimated that the self-consciousness of the patient, as to the origin and seat of her difficulties, as well as the opinions of previous medical advisers, must not be allowed to divert the physician from such a full and critical investigation, as in his opinion each and every case demands.

These propositions granted, it will be proper to speak, at a future time, of the natural supports of the uterus, their failure in office, and of the qualities necessary to be found in a pro perly adapted artificial support.

